# Dysregulated Hematopoietic Stem and Progenitor Cell Activity Promotes Interleukin-23-Driven Chronic Intestinal Inflammation

**DOI:** 10.1016/j.immuni.2012.08.025

**Published:** 2012-12-14

**Authors:** Thibault Griseri, Brent S. McKenzie, Chris Schiering, Fiona Powrie

**Affiliations:** 1 Translational Gastroenterology Unit, Experimental Medicine Division Nuffield Department of Clinical Medicine, University of Oxford, John Radcliffe Hospital, Oxford OX3 9DU, UK; 2 CSL Ltd. Research Department, Bio21 Molecular Science and Biotechnology Institute, University of Melbourne, 30 Flemington Road, Parkville, Victoria 3010, Australia; 3 Sir William Dunn School of Pathology, University of Oxford, Oxford OX1 3RE, UK

## Abstract

In interleukin-23 (IL-23)-dependent colitis, there is excessive accumulation of short-lived neutrophils and inflammatory monocytes in the intestine. It is unknown whether this reflects changes in mature cell populations or whether the IL-23-driven colitogenic T cell program regulates upstream hematopoietic stem and progenitor cells (HSPC). Here we have shown dysregulation of hematopoiesis in colitis mediated by inflammatory cytokines. First, there was an interferon-gamma-dependent accumulation of proliferating hematopoietic stem cells in the bone marrow and spleen. Second, there was a strong skew toward granulocyte-monocyte progenitor (GMP) production at the expense of erythroid and lymphoid progenitors. Extramedullary hematopoiesis was also evident, and granulocyte macrophage-colony stimulating factor (GM-CSF) blockade reduced the accumulation of splenic and colonic GMPs, resulting in amelioration of colitis. Importantly, transfer of GMPs exacerbated colitis. These data identify HSPCs as a major target of the IL-23-driven inflammatory axis suggesting therapeutic strategies for the treatment of inflammatory bowel disease.

## Graphical abstract

**Graphical Abstract:**

**Figure:**
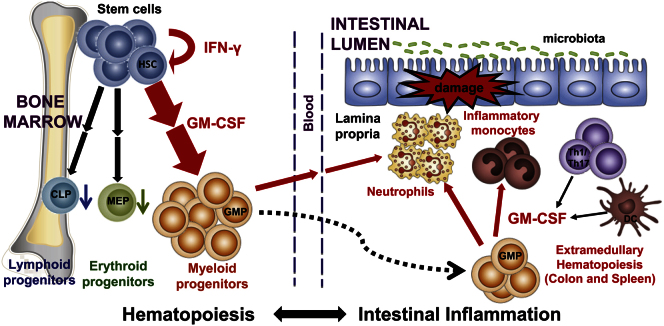

**Highlights:**

► Hematopoiesis is skewed toward the granulocyte/monocyte lineage during colitis ► Highly proliferative myeloid progenitors accumulate in the spleen and inflamed colon ► GM-CSF controls extramedullary hematopoiesis and promotes colitis ► Peripheral granulocyte/monocyte progenitors contribute to the pathogenesis of colitis

## Introduction

Dysregulated interleukin-23 (IL-23)-dependent responses have been shown to mediate experimental colitis in mice and have been linked to inflammatory bowel disease (IBD) in humans (Izcue et al., 2009). Recent evidence suggests that IL-23 acts as a molecular switch to promote pathological STAT3 and RORγt transcription factor-dependent T cell and innate lymphoid cell (ILC) responses (Ahern et al., 2010; Buonocore et al., 2010). Both T cell and ILC-driven colitis are characterized by marked accumulation of myeloid cells in the colon, particularly neutrophils and inflammatory monocytes (Griseri et al., 2010; Maloy et al., 2003; Erdman et al., 2009). Despite strong evidence of the pathophysiological role of IL-23 in colitis, little is known about the key downstream colitogenic mechanisms, particularly the link to increased myeloid cell responses.

Upon activation, neutrophils can release a plethora of inflammatory products (e.g., tumor necrosis factor [TNF], chemokines, proteases, and reactive oxygen species), and their excessive accumulation causes tissue damage in chronic inflammatory diseases (Nathan, 2006). In IBD patients, accumulation of tissue toxic neutrophils in the inflamed tissue correlates with clinical disease activity (Chin and Parkos, 2006) and the amount of fecal calprotectin (a predominant component of the neutrophil cytosol) is a clinical marker in IBD (Foell et al., 2009). In addition, high numbers of inflammatory monocytes and macrophages are present in the inflamed intestine in Crohn's disease patients (Kamada et al., 2008). To date, most therapeutic strategies in IBD have focused on targeting effector T cells. By contrast, the regulation of innate cell accumulation in the inflamed intestinal mucosa is poorly understood, particularly how inflammatory myeloid cells are regulated at their site of production, the bone marrow (BM). Chronic and sustained accumulation of myeloid cells places substantial pressure on granulomonocytopoiesis in the BM. Indeed, innate cells, especially neutrophils, have a very short life span compared to long-lived T and B cells, and tight regulation between supply from the BM and demand in the periphery is essential. However, the regulation of the upstream and primordial hematopoietic stem and progenitor cells (HSPCs) during colitis and other chronic inflammatory diseases is poorly characterized.

The development of HSPCs at steady state follows a hierarchy tightly regulated by endogenous signals, and the diverse steps of this developmental pathway can be precisely identified by flow cytometry, from the multipotent and self-renewing long-term hematopoietic stem cells (LT-HSCs) to the downstream and highly proliferative lineage-committed progenitors, e.g., the granulocyte-monocyte progenitors (GMPs) (see Figure S1A available online) (Kondo et al., 2003). Recent studies have shown that HSPCs are far more sensitive to exogenous environmental cues than previously anticipated, due in part to the expression of receptors for various microbial products and inflammatory cytokines, such as toll-like receptors (TLRs) and receptors to type I and type II interferons (IFNs) (Baldridge et al., 2010; Nagai et al., 2006; Takizawa et al., 2012). Despite our knowledge of the developmental process during the steady state, little is known about the hematopoietic control mechanisms during acute infection and even less in chronic inflammatory conditions (Takizawa et al., 2012).

Here we have asked how HSCs and progenitor cells are regulated during intestinal inflammation to determine whether they contribute to the pathophysiological network that drives colitis. Our results provide the evidence that HSC activity is dramatically modified during intestinal inflammation. There was a marked increase in HSC proliferation, and these cells accumulated not only in the BM of colitic mice but also at extramedullary sites. This was accompanied by a substantial skewing of HSC differentiation toward GMPs, which were increased in the BM as well as the inflamed spleen and colon. The inflammatory cytokines IFN-γ and GM-CSF promoted dysregulated hematopoiesis acting at distinct points in stem and progenitor cell differentiation. Furthermore adoptive transfer of splenic GMPs led to aggravation of colitis, providing a direct link to disease pathogenesis.

## Results

### Differential Regulation of Hematopoietic Lineages in Colitis

**Figure 1:**
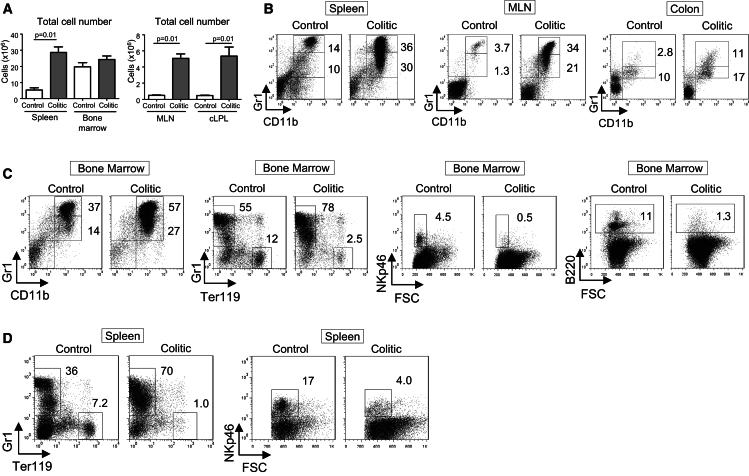
Differential Regulation of Hematopoietic Lineages in Colitis Colitis was induced by transfer of naive CD4^+^CD45RB^hi^ T cells into C57BL/6.*Rag1*^*−/−*^ mice. Colitic mice were killed 8 wk after transfer (termed colitic) and compared with untransferred *Rag1*^*−/−*^ mice (termed control). (A) Total cell numbers in spleen, bone marrow (one leg, femur and tibia), MLN, and colon (colonic lamina propria leukoctyes, cLPL) (±SEM, n = 5 mice per group). (B) Representative flow cytometry staining of neutrophils (CD11b^+^Gr1^hi^SSC^hi^) and inflammatory monocytes (CD11b^+^Gr1^int^SSC^lo^) from spleen, MLN, and colon (cLPL) cell suspensions. (C) Representative staining of neutrophils, monocytes, NK cells (NKp46^+^), erythroid cells (Ter119^+^), and immature B cells (B220^int^) from bone marrow cell suspensions. (D) Representative Gr1, Ter119, and NKp46 staining from spleen cell suspensions. Data are representative of three independent experiments.

To investigate the link between IL-23-driven pathogenic T cell responses and myeloid cell accumulation in colitis, we used a T cell transfer model that resembles aspects of IBD in humans. In this model, *Rag1*^−*/*−^ mice transferred with naive CD4^+^CD25^−^CD45RB^hi^ T cells develop wasting disease and colitis (Powrie et al., 1994) with splenomegaly, mesenteric lymph node (MLN) enlargement, and severe leukocytic infiltration of the colon (Figure 1A). In addition to accumulation of colitogenic T helper-1 (Th1) and Th17 cells (Griseri et al., 2010; Ahern et al., 2010), there was a marked increase of neutrophils (CD11b^+^Gr1^hi^SSC^hi^) and inflammatory monocytes (CD11b^+^Gr1^int^SSC^lo^) in the spleen, MLN, and colon 2 months after T cell transfer (Figure 1B). Analysis of mature cells of different lineages in the BM showed a similar increase in monocytes and neutrophils accompanying intestinal inflammation (Figure 1C). In parallel, the percentages of both lymphoid and erythroid populations, including natural killer (NK) cells, immature B cells (B220^int^), and Ter119^+^ erythroid cells, were reduced in the BM of colitic mice compared to controls (Figure 1C). These hematopoietic lineage changes in the BM were reflected in reduced percentages of NK cells and erythroid cells in the spleen of colitic mice and a considerable expansion of Gr1^+^ cells (Figure 1D).

### Increase in Proliferative HSC in the BM of Colitic Mice

**Figure 2:**
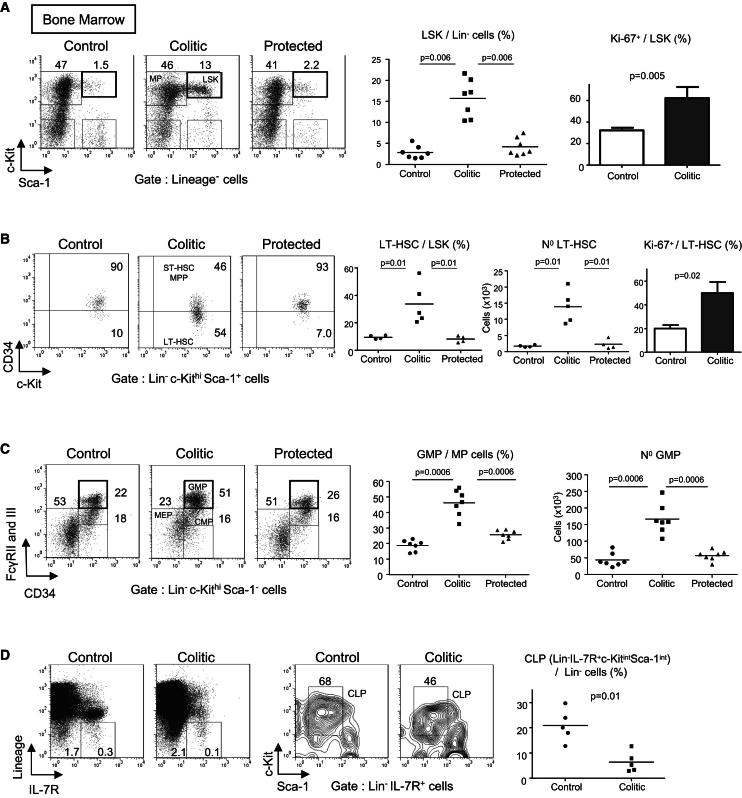
Increase in Proliferative HSCs in the Bone Marrow of Colitic Mice and Skewed Differentiation toward GMPs *Rag1*^−/−^ mice received either naive T cells alone (termed colitic) or in combination with CD4^+^CD25^+^ Treg cells (termed protected) or were untreated (termed control). Transferred mice were killed 8 wk after transfer. BM cells were stained for HSPCs (see Figure S1 for population FACS-definitions). (A) Representative flow cytometry staining and percentages of LSKs (Lin^−^Sca-1^+^c-Kit^hi^ cells, highly enriched for HSC) among BM Lin^−^ cells. In some experiments, BM cells were stained for the proliferative marker Ki-67 (right panel). (B) Percentages, absolute numbers, and proliferation of LT-HSCs (CD34^−^LSKs) in the BM. (C) Representative flow cytometry staining of CMP, GMP, and MEP population in the BM (left). Absolute numbers of GMPs (right) and percentages among Lin^−^Sca-1^−^c-Kit^hi^ MP cells (middle). (D) Flow cytometry staining of IL-7R expression among Lin^−^cells in the BM (left) and c-Kit and Sca-1 expression among Lin^−^IL-7R^+^ cells (middle). Percentages of CLP (Lin^−^IL-7R^+^Sca-1^int^c-Kit^int^) among Lin^−^ cells (right). Numbers in dot plots indicate percentages among gated cells. Each point represents an individual mouse and horizontal bars represent group means (A–D). Data are representative of three independent experiments. Error bars represent SEM.

As we observed substantial qualitative changes in mature cell composition within the BM, we next investigated whether the upstream HSPCs were affected by intestinal inflammation. Mature cell changes in the BM could be the result of modifications in proliferation of immature and precursor cells (lineage positive) or survival of mature cells with only a modest contribution from the stem and progenitor compartment. On the other hand, an uncontrolled increase of clonogenic HSPCs could have considerable repercussions on the severity and chronicity of intestinal inflammation. HSPCs reside in the IL-7R^−^ and IL-7R^+^ lineage (Lin) negative compartment (CD3^−^CD4^−^CD8^−^CD11b^−^CD11c^−^Gr1^−^Ter119^−^B220^−^NKp46^−^FcεRI^−^) that represents ∼2% of total BM cells (Figure 2D, left panel). Hematopoietic stem cells (HSCs) are rare cells, representing only ∼0.01% of total BM cells, but are highly enriched among Lin^−^Sca1^+^c-Kit^hi^ cells, designated as LSKs ([Kondo et al., 2003]; see Figure S1A). The inflammatory environment associated with colitis led to a 5.7-fold increase in the percentage of LSKs among Lin^−^ cells in the BM compared to control mice (Figure 2A) with a doubling in the percentage of proliferating LSKs (Ki-67^+^, Figure 2A).

LT-HSCs, the only life-long self-renewing HSCs, are enriched among CD34^−^LSK cells [(Osawa et al., 1996) and (Figure S1A)]. On average, 20% of LT-HSCs were cycling at steady state, whereas this number reached 50% during colitis, correlating with an 8-fold increase in the absolute number of LT-HSC in the BM of colitic mice (Figure 2B). Notably, protection from colitis by cotransfer of regulatory T cells (Treg cells), which are enriched among CD4^**+**^ T cells in the BM (Griseri et al., 2010), prevented the HSC expansion (Figures 2A and 2B). The use of the SLAM molecule CD150 as an additional marker for LT-HSCs (Kiel et al., 2005) confirmed the marked increase of LT-HSCs during intestinal inflammation (Figure S1B). Overall, HSC proliferation was increased in the BM of colitic mice, and LT-HSC accumulation was particularly striking, showing that the inflammation associated with colitis had a substantial impact on the most primordial steps of hematopoiesis.

### Intestinal Inflammation Skews HSC Differentiation toward GMPs

HSCs give rise to multipotent progenitors (MPPs) that have lost self-renewal capacity and will later differentiate into highly proliferative lineage-committed progenitors: either common myeloerythroid progenitors (CMPs) or common lymphoid progenitors (CLPs) (Kondo et al., 2003). CMPs will segregate into either megakaryocyte-erythroid progenitors (MEPs) or granulocyte-monocyte progenitors (GMPs) (Akashi et al., 2000; see Figure S1A for definitions). During colitis, the cell composition inside the myeloid progenitor population (MP, Lin^−^Sca-1^−^c-Kit^hi^) changed considerably. Thus the percentage of GMPs among MPs increased from 19% to 46% with a 3.9-fold change in total number in colitic mice (Figure 2C). This GMP increase, which was not observed in Treg cell protected mice, was mirrored by a substantial decrease in MEPs in the BM of colitic mice (Figure 2C, left panel). Lymphoid progenitors are Lin^−^ IL-7R^+^, whereas MPs are IL-7R^−^ (Kondo et al., 2003). During colitis, Lin^−^IL-7R^+^ cell frequency among Lin^−^ cells was reduced (Figure 2D) and the percentage of CLPs (Lin^−^IL-7R^+^Sca-1^int^c-Kit^int^) was significantly decreased compared to control mice (Figure 2D). Collectively, the progenitor composition inside the BM was dramatically altered during intestinal inflammation with a skew toward GMP differentiation, at the expense of both MEP and CLP differentiation.

A decrease in lymphoid cells was not restricted to *Rag1*^−/−^ hosts because lymphoid cell production in the BM was also decreased in a lymphocyte-replete model of colitis in which WT mice infected with *Helicobacter hepaticus* develop intestinal inflammation in the absence of IL-10 signaling (Kullberg et al., 2006). The large population of B cells (B220^+^) present in the BM of WT controls was considerably reduced during colitis as were erythroid cells (Figure S2A), in contrast with increases of Gr1^+^ myeloid cells in BM and spleen (Figures S2A and S2B). These mature cell changes also correlated with a significant increase of GMPs and decrease of MEPs and CLPs in the BM of lymphocyte-replete colitic mice (Figures S2C and S2D).

### Accumulation of HSCs and GMPs in the Spleen of Colitic Mice

**Figure 3:**
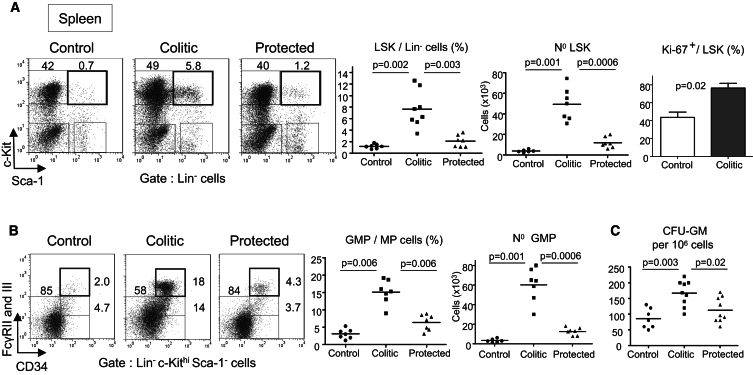
Accumulation of HSC and GMP in the Spleen of Colitic Mice *Rag1*^*−/−*^ mice received either naive T cells alone (colitic) or in combination with Treg cells (protected) or were untreated (control). Transferred mice were killed 8 wk after transfer. (A) Representative flow cytometry staining, percentages, absolute numbers, and proliferation (Ki-67) of LSK cells in the spleen. (B) Flow cytometry staining of splenic CMPs, GMPs, and MEPs. Percentages (middle) and numbers of splenic GMP (right). (C) Cfu activity. Frequency of clonogenic cfu-GM (cfu-granulocyte and macrophage) among total splenocytes. Data are representative of three independent experiments. Error bars represent SEM.

The low basal migration of HSCs from the BM to the periphery is a well-established phenomenon, but its functional relevance is unknown (Kondo et al., 2003). However, peripheral HSCs may contribute to emergency extramedullary hematopoiesis within infected tissues (Massberg et al., 2007). The rare population of HSCs in the blood and spleen resembles marrow HSCs because they are capable of long-term multilineage reconstitution after transfer into irradiated hosts (Bhattacharya et al., 2009; Morita et al., 2010). In the spleen of colitic mice, the percentage of proliferating LSKs was significantly higher than in controls, accompanied by a substantial accumulation of splenic LSKs (Figure 3A). In addition, the CD150^+^ population of LT-HSC that was very rare in the spleen of control mice was readily detectable in colitic mice (Figure S1B). Similarly, GMPs that were barely detectable in the spleen at steady state (Figure 3B) were significantly increased during colitis (4.9-fold in percentage and 20-fold in absolute number), whereas Treg cell protected mice showed only a modest GMP increase compared to controls (Figure 3B).

To determine the functional role of these changes, we assessed hematopoietic activity by quantifying colony forming units (cfu). In the spleen, the cfu-granulocyte and macrophage (cfu-GM) activity per cell was significantly increased in colitic mice compared to controls (Figure 3C). Because GMPs and mobilized HSCs have high proliferative indexes (Passegué et al., 2005), their accumulation in the inflamed spleen constitutes a reservoir of HSPCs capable of engaging in extramedullary hematopoiesis, which may contribute substantially to the increase of peripheral neutrophils and monocytes that promotes intestinal inflammation.

### GMPs Accumulate in the Inflamed Intestinal Mucosa and Trigger Extramedullary Hematopoiesis

**Figure 4:**
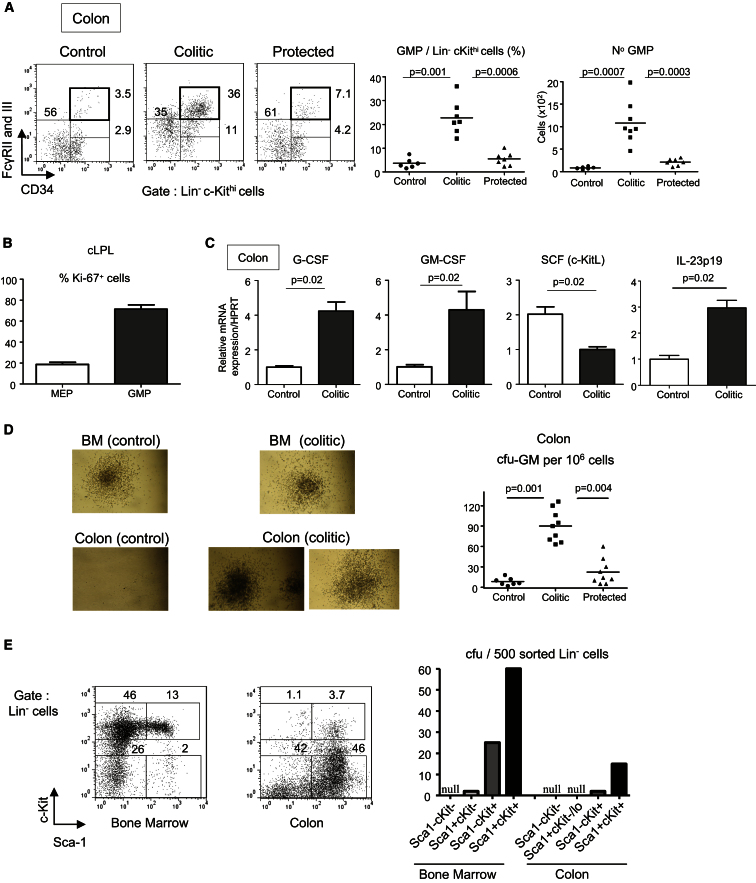
GMPs Accumulate in the Inflamed Intestinal Mucosa and Trigger Extramedullary cfu Activity in the Colon cLPL were prepared from control, colitic, and protected mice. Transferred mice were killed 8 wk after T cell transfer. (A) Representative CMP, GMP, and MEP staining in cLPL. Percentage and absolute number of colonic GMPs. (B) Percentages of proliferative cells (Ki-67^+^) among MEPs and GMPs from cLPL of colitic mice. (C) G-CSF, GM-CSF, SCF (c-KitL), and IL-23p19 mRNA amounts in total colon homogenate (n = 4). Values are normalized to HPRT and are mean values (±SEM). (D) Cfu activity from plated cLPL. Representative field from photomicrographs of Methocult plate with typical images of cfu (magnification ×10). Frequency of cfu-GM among total cLPL (right). (E) Representative c-Kit and Sca-1 staining gated on Lin^−^ cells from BM and cLPL of colitic mice (left). Number of cfus from 500 plated Lin^−^ cells isolated by cell sorting from BM or cLPL of 5 colitic mice; the various Lin^−^ FACS-sorted populations are identified on the horizontal axis (right). (A–D) Data are representative of two to three independent experiments.

Because the colonic increase of neutrophils and monocytes is extensive and sustained over the course of colitis (Figures 1A and 1B), we investigated whether peripheral HSPC activity could be detected in the inflamed intestinal mucosa. Colonic GMPs were barely detectable at steady state, but their frequency was markedly increased in colitis (Figure 4A) with an accompanying 13.5-fold rise in absolute number compared to controls (Figure 4A). GMPs also emerged in the inflamed MLN (Figure S3A). It is important to note that, as previously described in BM and spleen (Passegué et al., 2005), the GMP population was highly proliferative in the colon (Figure 4B) and therefore had the potential to generate neutrophils and monocytes directly inside the inflamed tissue. Consistent with this, the amounts of messenger RNA (mRNA) for granulocyte-colony stimulating factor (G-CSF) and granulocyte and macrophage colony stimulating factor (GM-CSF) (the key colony stimulating factors [CSF] that regulate granulocyte and monocyte progenitors) were increased in the inflamed colon and therefore capable of sustaining proliferation of colonic GMPs (Figure 4C).

Functionally, the phenotypic changes in progenitors in the inflamed colon correlated with striking modifications in hematopoietic activity. Indeed, cfu-GM activity per cell, which was very low in the colon of control mice (Figure 4D), was substantially increased in the colon of colitic mice, equating to a 12-fold rise of cfu-GM, with no significant increase in Treg cell protected mice (Figure 4D). Cell sorting experiments showed that the intestinal cfu activity evident in colitis arose from the same rare cellular subtypes as in the BM, i.e., Lin^−^ cells expressing high amounts of c-Kit (Figure 4E). Other colonic Lin^−^ populations, i.e., Lin^−^Sca-1^−^c-Kit^−^ and Lin^−^Sca-1^+^c-Kit^−/int^ cells that are both predominant in the colon, did not exhibit cfu activity (Figure 4E). The LSK population that was observed in the inflamed colon (Figure 4E) did not contain CD150^+^ LT-HSC, but was CD34^+^CD150^−^, correlating with a MPP phenotype (Figure S3B).

These results suggest that the increase of inflammatory myeloid cells in the colon following colitogenic Th1 and Th17 cell-mediated responses may be partially fueled by colonic HSPC accumulation. Of particular relevance are highly proliferative GMPs that contribute to increased cfu activity and by extension to extramedullary granulomonocytopoiesis during intestinal inflammation.

### Extramedullary Accumulation of GMPs Is Dependent on Local Tissue Inflammation

**Figure 5:**
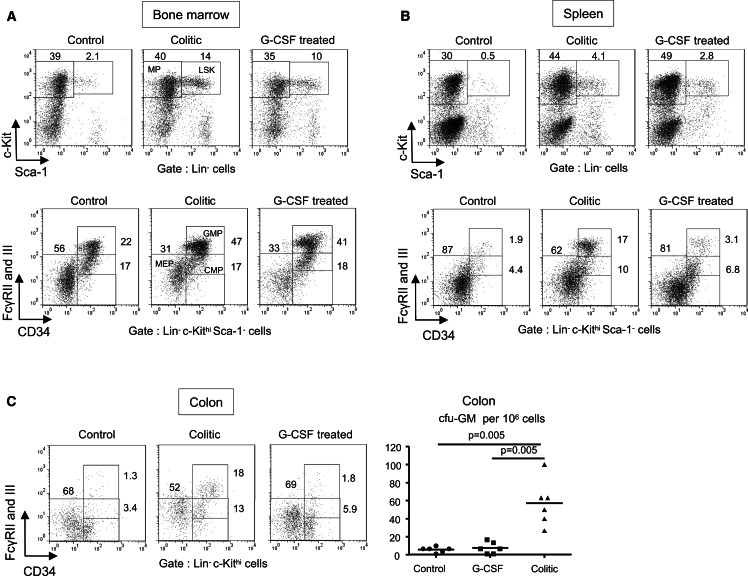
Extramedullary Accumulation of GMPs during Colitis Is Dependent on Local Tissue Inflammation C57BL/6.*Rag1*^*−/−*^ mice were injected daily with G-CSF for 3 days and killed 24 hr after the last G-CSF injection (termed G-CSF treated) and compared with *Rag1*^−/−^ mice killed 8 wk after CD4^+^CD45RB^hi^ T cell transfer (colitic) and with untransferred *Rag1*^−/−^ mice (control). (A and B) Representative flow cyometry staining of LSKs (upper panels) and GMPs (lower panels) in the BM (A) and the spleen (B) (individual mice, n = 4). (C) Representative flow cytometry staining of GMPs in colonic LPL is shown on left (n = 4). Colonic cfu-GM activity is shown on right. Data are representative of two independent experiments.

Because HSCs and GMPs accumulated in the BM and periphery during colitis, we tested whether injection of G-CSF would recapitulate the phenotype observed during colitis. Indeed, G-CSF promotes granulopoiesis and is also a prototypical mobilizing cytokine, stimulating proliferation of HSCs in the BM and their release into the blood (Morrison et al., 1997). Treatment with G-CSF, a cytokine that was increased in colitis (Figure 4C), was sufficient to promote an increase in LSKs and GMPs in the BM (Figure 5A). In the spleen, G-CSF induced an accumulation of recirculating LSKs comparable to colitis, but only a modest GMP increase (Figure 5B). Importantly, there was no accumulation of GMPs detected in the colon of G-CSF treated mice and the cfu-GM activity was as low as in control mice (Figure 5C). Similarly, treatment with GM-CSF stimulated GMP increase in the BM but did not induce any colonic GMP accumulation (Figure S4). These results show that the colonic and splenic accumulation of GMPs accompanying colitis cannot be reproduced by a simple mobilization protocol and likely requires additional local tissue inflammatory signals associated with IL-23-driven colitis.

### IFN-γ Promotes Increased HSC Activity during IL-23-Driven Colitis

**Figure 6:**
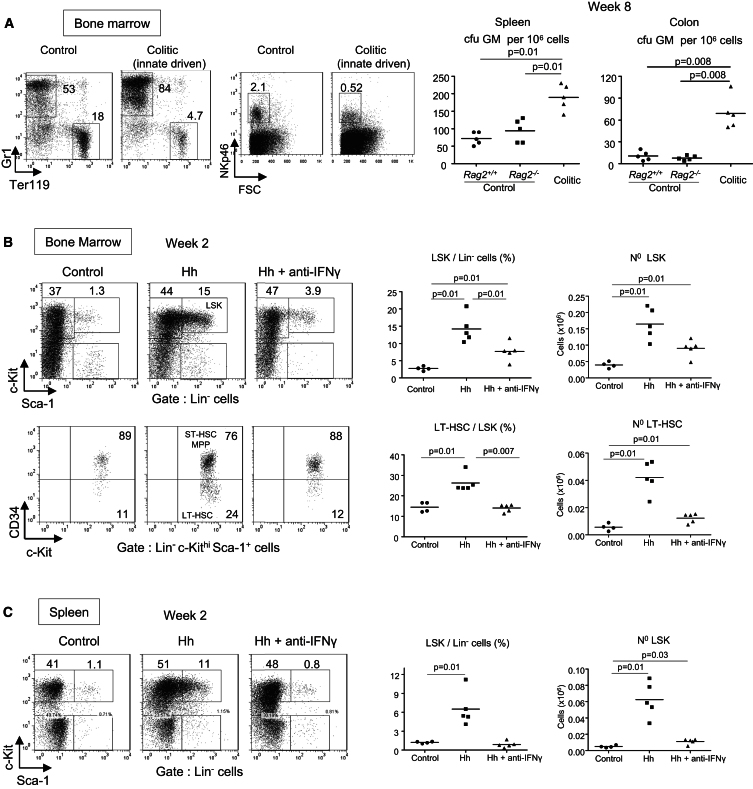
IFN-γ Promotes Exacerbated HSC Activity during IL-23-Driven Colitis Innate colitis was induced by *H. hepaticus* (Hh) infection of 129SvEv *Rag2*^−/−^ mice. Mice were killed 8 wk (colitic) in (A) or 2 wk (Hh) in (B) and (C) after *H. hepaticus* feeding and compared to untreated *Rag2*^*−/−*^ mice (control) or untreated *Rag2*^*+/+*^ mice where indicated. (A) Representative Gr1, Ter119, and NKp46 staining in the BM is shown on left. Frequency of clonogenic cfu-GM among total splenocytes and cLPL is shown on right. (B) Infected mice were either injected weekly with anti-IFN-γ (Hh + anti-IFN-γ) or left untreated (Hh). Flow cytometry analysis and percentages of LSKs in BM (upper panels). Percentages and absolute numbers of LT-HSCs in BM (lower panels). (C) FACS analysis, percentages, and absolute numbers of LSKs in spleen. (A–C) Data are representative of two independent experiments.

We next investigated which inflammatory cytokines drive the hematopoietic changes associated with IL-23 dependent intestinal inflammation. IFN-γ is a well-known colitogenic cytokine that is increased in the colon and serum of colitic mice (Powrie et al., 1994; Ahern et al., 2010), and its secretion by pathogenic Th1 and Th17 cells and ILCs is promoted by IL-23 (Ahern et al., 2010; Buonocore et al., 2010). Its receptor is not only expressed by mature immune cells, but also by HSCs, which are directly responsive to IFN-γ stimulation in vitro, as well as in vivo during both homeostasis and following infection (Baldridge et al., 2010). In addition to the role of IFN-γ in T cell mediated disease, IL-23 dependent innate colitis induced by *H. hepaticus* infection of 129SvEv.*Rag2*^−/−^ mice is inhibited by blockade of IFN-γ (Buonocore et al., 2010). In this innate model of colitis, Gr1^+^ myeloid cells accumulate in the colon and spleen and drive intestinal inflammation (Erdman et al., 2009; Maloy et al., 2003). A similar Gr1^+^ myeloid cell increase was observed in the BM of *H. hepaticus* infected mice (Figure 6A). As we observed in T cell dependent colitis, erythroid cells and NK cells were decreased compared to uninfected controls (Figure 6A). At the HSPC level, there was a significant rise in LSK and LT-HSC numbers in the BM and spleen (Figures S5A and S5B) and in cfu-GM activity in the colon and spleen 8 wk after infection (Figure 6A). In this T cell independent model of colitis, blockade of IFN-γ diminished LSK increases in the BM (Figure 6B) and spleen (Figure 6C), as well as CD34^−^ LT-HSC and CD150^+^ LT-HSC increases in the BM (Figure 6B; data not shown). Similar IFN-γ promoting effects on HSCs were observed in T cell transfer colitis (data not shown) in which a population of IFN-γ producing T cells accumulated not only in the spleen and colon (Ahern et al., 2010), but also in the BM (Figures S6E) where they may have increased HSC numbers directly. Interestingly, Treg cell protected mice in which HSC did not accumulate in the BM (Figure 2A) exhibited much lower numbers of IFN-γ^+^ T cells in BM than colitic mice (Figures S6E). These data, taken together with previous studies demonstrating cell-intrinsic IFN-γ responsiveness by HSC (Baldridge et al., 2010), suggest that IFN-γ may exert its pathogenic role in colitis not only by promoting effector functions in mature leukocytes but also by boosting the hematopoietic activity at the level of the primordial HSCs that sustain the increased production of downstream and non-self-renewing GMPs.

### GM-CSF Promotes Pathogenic Accumulation of Peripheral GMPs and IL-23 Dependent Colitis

**Figure 7:**
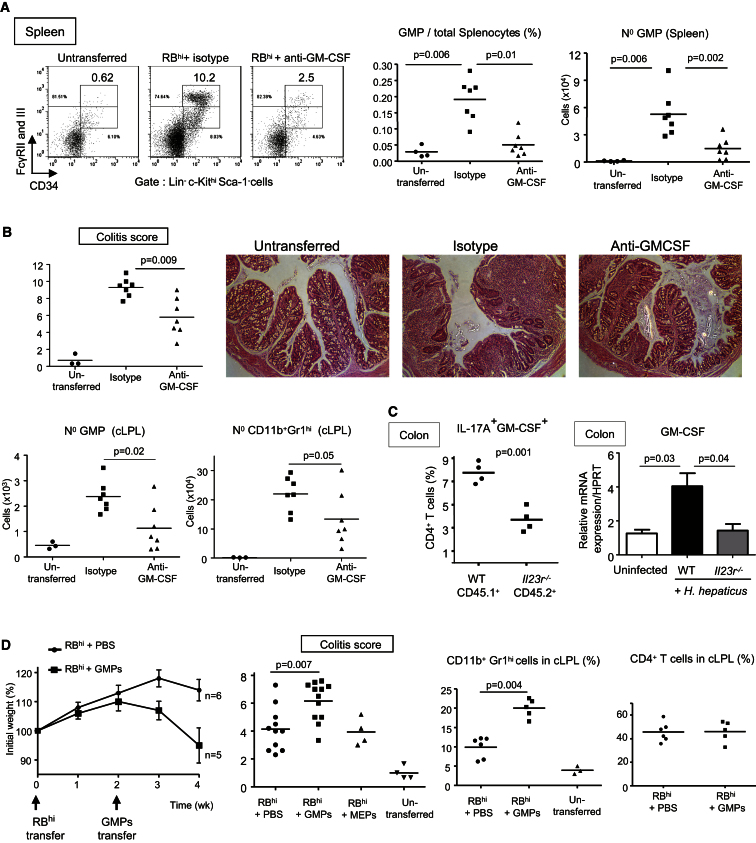
GM-CSF Promotes Pathogenic Accumulation of Peripheral GMPs and IL-23 Dependent Colitis (A and B) *Rag1*^−/−^ mice transferred with naive T cells were injected two times per week with either anti-GM-CSF mAb or isotype control, killed 8 wk after T cell transfer, and compared with untransferred control mice. (A) Representative staining, frequencies, and absolute numbers of GMPs in the spleen. (B) Score of colitis and photomicrographs of proximal-colon sections (upper panels). Original magnification 100×. GMP and neutrophil numbers in the cLPL (lower panels). (A and B) Data are representative of two independent experiments. (C) Left shows that *Rag1*^−/−^ mice were cotransferred with 1:1 mixtures of CD45.1^+^ (WT) + CD45.2^+^ (*Il23r*^−/−^) naive CD4 T cells to assess whether IL-23 has a cell intrinsic effect on GM-CSF production by Th cells as described in (Ahern et al., 2010). CD45.1^+^ cells were isolated from congenic B6.SJL-Cd45.1 and CD45.2^+^ cells from *Il23r*^−*/*−^ mice. Mice were killed 4 wk after T cell transfer and percentages of IL-17A^+^ and GM-CSF^+^ cells in colonic CD4^+^ T cells were assessed by intracellular flow cytometry. Right shows that WT and *Il23r*^−/−^ C57BL/6 mice were infected with *H. hepaticus* and treated with anti-IL10R. WT Mice were killed when colitic (week 2) and compared with *Il23r*^−/−^ protected mice and unmanipulated WT mice for GM-CSF mRNA amounts in total colon homogenate (n = 4). (D) *Rag1*^−/−^ mice that had received naive T cells (“RB^hi^”) 14 days before were injected intravenously with PBS or 1 × 10^4^ sorted splenic GMPs or MEPs, where indicated. GMPs and MEPs were sorted from the spleen of colitic mice (8 wk post-T cell transfer). Shown are weight loss and colitis score 14 days after PBS, GMPs, or MEPs injection and frequencies of neutrophils and CD4^+^ T cells in cLPL. Weight loss is representative of two independent experiments, and colitis score is pooled from two independent experiments. Error bars represent SEM.

Cytokines that drive myelopoiesis, such as the various CSFs, are produced by stromal cells (e.g., epithelial cells and fibroblasts) and by innate and adaptive immune cells. GM-CSF has been shown to be pathogenic in experimental autoimmune encephalomyelitis (EAE) and arthritis models (Hamilton, 2008) although its mechanism of action is not known. Here we show a clear role for GM-CSF in HSPC activity and development of colitis. Administration of anti-GM-CSF inhibited the peripheral accumulation of GMPs and attenuated intestinal inflammation in T cell transfer colitis (Figures 7A and 7B). GMP percentages and total numbers were significantly decreased in the spleen of T cell transferred mice injected with anti-GM-CSF compared to mice injected with an isotype control mAb (Figure 7A). In contrast, GM-CSF was not essential for the increase of the upstream HSCs in the spleen and BM (Figure S6A) and therefore regulates HSPC activity downstream of IFN-γ. In the colon, GMPs were also reduced after anti-GM-CSF treatment, and this was accompanied by decreased numbers of neutrophils and histologic inflammation in the colon (Figure 7B). Intestinal GM-CSF amounts were substantially increased in colitic mice compared to control and *Il23r*^*−/−*^ protected mice (Figures 4C and 7C). Transfer experiments utilizing congenically marked IL-23R^+^ and IL-23R^−^ T cells in which both populations accumulate in the colon similarly (Ahern et al., 2010) showed increased GM-CSF production among WT colitogenic Th17 cells (Figure 7C). Further analysis of IL-17A^+^GM-CSF^+^ cells showed the majority coexpressed IFN-γ, were enriched in the colon relative to the spleen, and were present at higher proportions in IL-23R-expressing cells (Figure S6C). However, it should be noted that polyfunctional Th17 cells are not the only source of GM-CSF and other T cell and non-T cell sources were also detectable (Figure S6C; data not shown). Analysis of the requirement for IL-23 in colitis-associated changes in extramedullary hematopoiesis showed a significantly reduced, although not completely absent, accumulation of GMP in the BM and periphery of T cell transferred *Rag1*^*−/−*^*Il23p19*^*−/−*^ mice that developed mild intestinal inflammation (Figure S6D; Hue et al., 2006). Collectively, our results show that GM-CSF is a key driver of extramedullary accumulation of GMPs in IL-23-dependent intestinal inflammation and contributes to the pathogenicity of the disease. (Figure S6D).

To assess the contribution of peripheral GMPs to intestinal inflammation, we transferred splenic GMPs into T cell-restored *Rag1*^*−/−*^ mice. Adoptive transfer of splenic GMPs isolated from colitic mice into *Rag1*^*−/−*^ mice that had received naive T cells 2 weeks before enhanced disease as indicated by an increased colitis score and weight loss (Figure 7D). The GMP-mediated aggravation of colitis correlated with a significant increase of CD11b^+^Gr1^hi^ cells in the spleen (data not shown) and colon (Figure 7D) compared to mice that had only received naive T cells and PBS, whereas percentages of CD4^+^ T cells were unchanged (Figure 7D). As anticipated, transfer of peripheral GMPs gave rise to CD11b^+^Gr1^+^ myeloid cells during intestinal inflammation (Figure S6B). Thus, in addition to contributing to increased granulopoiesis in the BM, GMPs also accumulate in the inflamed spleen and colon, where they play a pathogenic role likely by sustaining the local accumulation of neutrophils and inflammatory monocytes that is characteristic of IL-23-driven intestinal inflammation.

## Discussion

The development of pathogenic T cell responses during autoimmune and chronic inflammatory diseases and their escape from Treg control in target tissues has been extensively studied. However, the impact of these dysregulated mature cell responses on myeloid cell development is poorly understood. In this study, by investigating the regulation of hematopoiesis during chronic colitis, we provide evidence that IL-23-driven intestinal inflammation is accompanied by profound changes in both the hematopoietic progenitor cell and stem cell compartments and that these can contribute directly to disease pathogenesis. We further show that inflammatory cytokines IFN-γ and GM-CSF control distinct components of the aberrant hematopoietic response. Thus, IFN-γ promoted increased HSC numbers in the BM, along with extramedullary accumulation of HSCs in the spleen. By contrast, GM-CSF was involved in the accumulation of highly proliferative GMPs in the inflamed spleen and colon and was essential for development of colitis.

Remarkably, the hematopoietic hierarchy was affected by the ongoing intestinal inflammation as early as its most primordial stage because LT-HSCs were greatly increased in the BM during colitis. HSC proliferation was substantially increased during colitis and correlated with a sustained differentiation into GMPs and a subsequent increase of Gr1^+^ myeloid cells in the BM. Classically, HSCs are perceived as passive components of the immune response, replenishing available niches to maintain immune system homeostasis (Kondo et al., 2003). However, recent studies in the field of antimicrobial immunity show that HSC proliferation is directly stimulated by certain inflammatory cytokines and TLR ligands (Takizawa et al., 2012). For example, IFN-γ secretion upon *Mycobacterium avium* infection directly increases HSC proliferation and numbers in the BM (Baldridge et al., 2010). Our observations in colitis show that HSC activation in response to environmental cues is not solely restricted to antimicrobial responses but also occurs during noninfectious chronic inflammatory disease. However, it is important to highlight that HSC accumulation is not a general feature of inflammatory immune responses. Indeed, during *Plasmodium chabaudi* infection, an atypic myelolymphoid progenitor emerged in the BM but LT-HSCs were not increased (Belyaev et al., 2010). Furthermore, autoimmunity in *Foxp3*^−/−^ mice was associated with decreased marrow LT-HSC numbers during the course of disease (Chen et al., 2010). Indeed, the nature of the eliciting immune stimulus appears to shape the hematopoietic response. Thus, contrary to what we observed during colitis, GMPs were decreased but CLPs were increased in the BM of *M. avium*-infected mice (Baldridge et al., 2010). Immunity to the *Trichinella spiralis* helminth correlated with substantial accumulation of eosinophil progenitors in the BM of infected mice (Iwasaki et al., 2005), and the pro-Th2 cell-derived cytokine IL-25 elicited the specific production of Th2 cell-biased multipotent progenitors (MPP_2_) (Saenz et al., 2010). Thus, analogous to the specialized T helper cell responses that develop according to the nature of the microbial stimuli, functional specialization among HSPCs allows the hematopoietic system to mount a precise response adapted to the specific peripheral challenge.

In addition to profound changes in HSPCs in BM, HSCs and GMPs accumulated in the spleen during colitis. High numbers of undifferentiated monocytes reside in the spleen, outnumbering their equivalent in the blood, and these splenic monocytes rapidly migrate to tissues upon inflammation (Swirski et al., 2009). We also found that the inflamed spleen constitutes a reservoir of HSCs and GMPs that might have contributed to the increase in splenic myeloid cells and could also migrate to the colon during inflammation. Interestingly, *Foxp3*^−/−^ mice develop a splenomegaly that is accompanied by splenic cfu-GM increase, and this was inhibited by the infusion of CD25^+^ Treg cells at birth (Lee et al., 2009). In our study, protection from colitis by Treg cells prevented increased HSPC activity in the BM and periphery. It is possible that in addition to effects on mature immune cells, Treg cells directly inhibit HSPC activity via suppressive cytokine secretion. Indeed, key cytokines involved in Treg-mediated control of colitis have been shown to affect HSC, TGF-β directly signals to HSCs to restrain their proliferation (Yamazaki et al., 2009) and a large proportion of HSCs also express the IL-10R (Kang et al., 2007).

Increased hematopoietic activity in the spleen of colitic mice was accompanied by an unexpected cfu-GM activity in the inflamed colon that correlated with GMP accumulation. The α4β7 was expressed by 10% to 20% of HSPCs, and this colon homing receptor was increased on BM and splenic HSCs during colitis (Figure S3C). Its ligand MAdCAM1 is increased during intestinal inflammation (Souza et al., 1999); thus, HSPCs might be guided to the inflamed colon as a result of their expression of α4β7 integrin. At steady state, a rare but reproducible cfu activity was detectable in the colon in line with previous observations that a scarce population of recirculating HSPCs constantly survey nonlymphoid tissues and can trigger local urgent hematopoiesis upon TLR stimulation (Massberg et al., 2007). This mechanism, from which a sizable pool of myeloid cells originated, is potentially beneficial for the host during microbial infection but detrimental during chronic inflammation. Indeed, GMPs that accumulated in inflamed colon were highly proliferative and may provide a rapid and local source of myeloid cells that sustains disease. Indeed, adoptive transfer of peripheral GMPs aggravated ongoing colitis and correlated with increased CD11b^+^Gr1^+^ cells in colon and spleen.

With regard to therapeutic utility, it is clearly of interest to identify the various inflammatory cytokines produced during colitis that promote exacerbated HSC proliferation and biased differentiation toward GMPs. HSCs express receptors for IFNs, IL-1, and IL-6 (Chen et al., 2010; Ueda et al., 2009) and are therefore poised to respond promptly to various inflammatory stimuli. Recent studies have identified direct activity of IFN-γ on HSC. Thus, HSCs express high amounts of IFN-γ-R and proliferate in an IFN-γR1- and Stat1-dependent fashion, in response to *M. avium*-induced IFN-γ secretion (Baldridge et al., 2010). Control of HSC proliferation by IFN-γ also occurs under homeostatic conditions because injection of recombinant IFN-γ increased proliferation of LT-HSC in normal mice and there was reduced endogenous HSC proliferation in *Ifng*^−/−^ mice (Baldridge et al., 2010). In our study, IFN-γ blockade, which ameliorates colitis in both innate and T cell transfer models (Buonocore et al., 2010; Powrie et al., 1994), significantly reduced the BM and splenic accumulation of HSCs. In colitic mice, there was a marked accumulation of IFN-γ^+^ T cells in the BM where they may directly influence the HSC population. It was notable that the accumulation of IFN-γ^+^ T cells in BM was inhibited by Treg cell cotransfer but still occurred in the absence of IL-23 (Figure S6E). This activity correlates with modest IL-23 independent increases in LSK and GMP in the BM of T cell restored *Il23p19*^*−/−*^*Rag1* ^*−/−*^ compared to unmanipulated controls. This result fits with our previous observations of IL-23 independent systemic inflammation in T cell restored immune-deficient mice (Hue et al., 2006). These results suggest that IFN-γ may exert its colitogenic activity not only by promoting the effector arm of the immune system, but also by acting on the primordial step of the hematopoietic system by increasing HSC proliferation during colitis. Enhanced activity of the self-renewing HSC is probably crucial for the chronicity of increased myelopoiesis during colitis, because it allows for a sustained supply of short-lived medullary and peripheral GMPs (Passegué et al., 2005). HSC differentiation into various lineage-committed progenitors is influenced by cytokines acting on HSPCs downstream of IFN-γ, e.g., the various CSFs. For example, GM-CSF is thought to drive the HSCs away from CLP development in favor of CMPs (Kondo et al., 2003) and later promote both proliferation and survival of GMPs (Kimura et al., 2009).

Recently IL-23 dependent secretion of GM-CSF by T helper cells has been shown to be crucial in experimental allergic encephalomyelitis (EAE) development (Codarri et al., 2011; El-Behi et al., 2011). However the downstream effects of GM-CSF during chronic inflammatory diseases are still unclear. Among HSPC, GM-CSF-R is expressed by CMPs and GMPs, but absent from MEPs and CLPs (Takizawa et al., 2012). In colitis, neutralization of GM-CSF markedly decreased the splenic and colonic accumulation of GMPs and the severity of intestinal inflammation but had no effect on HSC numbers. In line with these observations, GM-CSF treatment induced GMP but not HSC increase in BM and spleen. Therefore, one of the pathogenic roles played by GM-CSF, which is secreted not only by Th cells but also by myeloid cells and stromal cells during inflammation (Hamilton, 2008), is to trigger extramedullary hematopoiesis and increase inflammatory myeloid accumulation in target organs during chronic inflammatory disease. In addition to promoting myeloid progenitor activity during colitis, GM-CSF may have additional effects on mature cells. Indeed, GM-CSF enhances IL-23 production by antigen-presenting cells in vitro (El-Behi et al., 2011). Those results together with our own suggest a positive feedback loop in which IL-23 stimulates GM-CSF production by polyfunctional Th17 cells in the colon, which in turn may act to further enhance IL-23 production by APC. Cytokine-driven alterations in hematopoiesis may be a common and somewhat overlooked pathogenic mechanism. For example, IL-17 has been reported to stimulate hematopoietic activity in the BM via stimulation of G-CSF secretion by stromal cells, and in the spleen via G-CSF dependent and independent mechanisms (Schwarzenberger et al., 2000).

Collectively, our results shed light on the selectivity and complexity of cytokine-mediated regulation of HSPC activity during IL-23-driven chronic inflammation. They support a “two hit” model for aberrant intra- and extramedullary myelopoiesis in colitis as follows: (1) IFN-γ-dependent increased HSC activity followed by (2) GM-CSF-dependent increased HSC differentiation into highly proliferative GMPs. Ultimately, we believe that precisely dissecting the cytokines and microbial signals that control HSPC function will offer opportunities to develop novel therapeutics in IBD and other chronic inflammatory diseases with a strong myeloid component (e.g., rheumatoid arthritis) and will also improve our general understanding of the regulation of hematopoiesis.

## Experimental Procedures

### Mice

Wild-type C57BL/6, congenic B6.SJL-Cd45.1, C57BL/6.*Rag1*^*−/−*^, C57BL/6.*Rag1*^*−/−*^*Il23p19*^*−/−*^, C57BL/6.*Il23r*^*−/−*^, 129SvEv, and 129SvEv.*Rag2*^*−/−*^ mice were bred and maintained under specific pathogen–free conditions in accredited animal facilities at the University of Oxford. Where indicated, mice were intraperitoneally (i.p.) injected with recombinant hG-CSF (5 μg /injection; CSL Ltd) or mGM-CSF (5 μg/injection; Peprotech) for three consecutive days and were killed 24 hr after the last injection. All procedures involving animals were conducted according to the requirements and with the approval of the UK Home Office Animals (Scientific Procedures) Acts, 1986. Mice were negative for *Helicobacter* spp. and other known intestinal pathogens and were 8–12 weeks old when first used.

### Induction of Colitis

For the T cell transfer model of colitis, naive CD4^+^CD25^−^CD45RB^hi^ T cells were FACS-sorted from C57BL/6 mice, as described previously (Izcue et al., 2008) and injected i.p. into C57BL/6.*Rag1*^*−/−*^ recipients (4 × 10^5^/mouse). Protective CD4^+^CD45RB^lo^CD25^+^ Treg cells (2 × 10^5^/mouse) were coinjected i.p. where indicated. Any mice losing >20% of their starting body weight or showing severe signs of disease were killed. Where indicated, mice were i.p. injected two times per week with 0.25 mg of anti-GM-CSF (MP1-22E9; CSL Ltd) or isotype control (GL117, rat IgG2a), starting from the first day of the T cell transfer. Induction of colitis in *H. hepaticus* dependent models is detailed in the Supplemental Experimental Procedures.

### Histological Assessment of Intestinal Inflammation

Proximal, mid-, and distal colon samples were fixed in buffered 10% formalin solution. Paraffin embedded sections were cut (5 μm) and stained with hematoxylin and eosin, and inflammation was scored in a blinded fashion using a previously described scoring system (Izcue et al., 2008).

### Isolation of Leukocyte Subpopulations and Flow Cytometry

Single cell suspensions were prepared from spleen, MLN, and cLPL as previously described (Uhlig et al., 2006). BM cell suspensions were prepared by flushing the marrow out of femur and tibia and were resuspended in PBS with 2% BSA. Monoclonal antibodies used for flow cytometry analysis of mature leukocytes and HSPCs are detailed in the Supplemental Experimental Procedures.

### Colony Forming Assay

ACK lysed splenocytes (1 × 10^5^ cells), cLPL (3 × 10^5^ cells), or flow cytometry-purified LSK or Lin^−^Sca-1^−^cKit^hi^ cells (500 cells) were suspended in methylcellulose medium to quantify clonogenic colony forming units in triplicate cultures (MethoCult GF M3434, Stem Cell Technologies). This medium contains IL-3, IL-6, and SCF but is G-CSF- and GM-CSF-free. Morphologic analysis of colony formation was performed after 10–14 days of incubation at 37°C using an inverted microscope. cfu-G (granulocyte), M (macrophage), or GM (granulocyte-macrophage) colonies are referred as “cfu-GM.”

### Quantitation of Gene Expression by Using Real-Time PCR

After homogenization of frozen colonic samples, total tissue RNA was purified using RNAeasy kits (QIAGEN), and quantitative PCR were performed as described in the Supplemental Experimental Procedures.

### Statistical Analysis

Statistical analysis was performed with Prism 5.0 (GraphPad Software). The nonparametric Mann-Whitney test was used for all statistical comparisons. Differences were considered statistically significant when p < 0.05.

## Supplemental Information

Document S1. Figures S1–S6 and Supplemental Experimental Procedures
