# Supplementary material for: Dysregulated Hematopoietic Stem and Progenitor Cell Activity Promotes Interleukin-23-Driven Chronic Intestinal Inflammation
Source: Immunity. 2012 Dec 14;37(6):1116–29. doi: 10.1016/j.immuni.2012.08.025 (PMC3664922; doi:10.1016/j.immuni.2012.08.025)
Supplement: Document S1. Figures S1–S6 and Supplemental Experimental Procedures [file mmc1.pdf]

Immunity, Volume *37*

Supplemental Information

Dysregulated Hematopoietic Stem and Progenitor Cell Activity Promotes Interleukin-23-Driven Chronic Intestinal Inflammation

Thibault Griseri, Brent S. McKenzie, Chris Schiering, and Fiona Powrie

Supplemental Inventory

1. Supplemental Figures and Tables

Figure S1, Related to Figure 1

Figure S2, Related to Figure 2

Figure S3, Related to Figure 4

Figure S4, Related to Figure 5

Figure S5, Related to Figure 6

Figure S7, Related to Figure 7

2. Supplemental Experimental Procedures

Figure S1, Related to Introduction and Figure 1

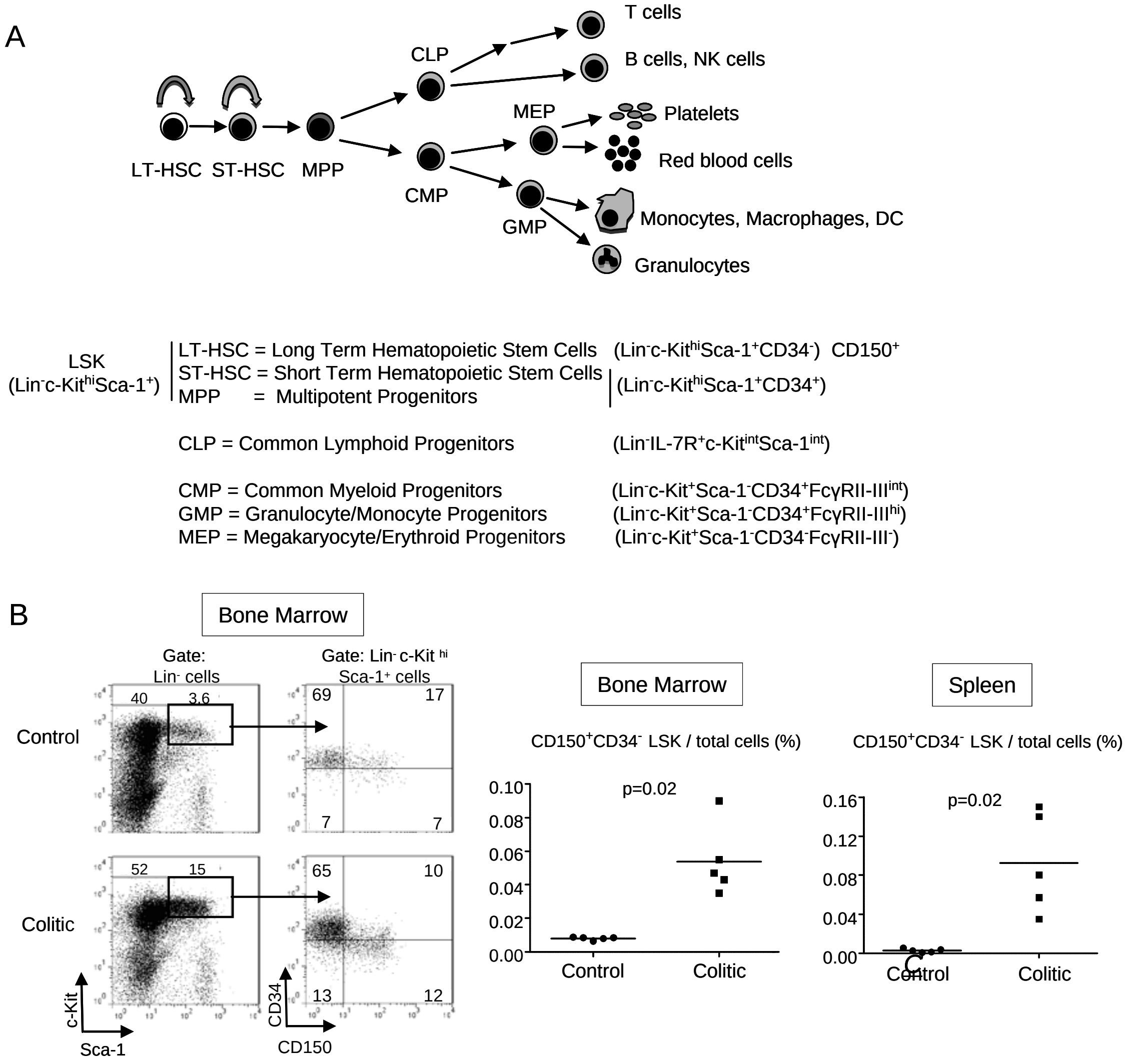

Figure S1. Schematic of Hematopoietic Development and Identification of LT-HSC by CD150 Expression, Related to Figure 1

(A) Schematic of hematopoietic development and cell-surface phenotype of hematopoietic stem cells and progenitor cells (HSPCs).

(B) Colitis was induced by transfer of 0.4×10^6 $CD4^+CD45RB^{hi}$ T cells into C57BL/6.*Rag1*$^{-/-}$ mice. Colitic mice were killed 8 weeks after transfer (termed colitic) and compared with untransferred *Rag1*$^{-/-}$ mice (termed control). Percentages of $CD150^+CD34^-$LSK (LT-HSC) among total BM cells and splenocytes are depicted. Data are representative of two independent experiments, each point represents an individual mouse and horizontal bars represent group means. Statistical significance was determined using a Mann Whitney test.

Figure S2, Related to Figure 2

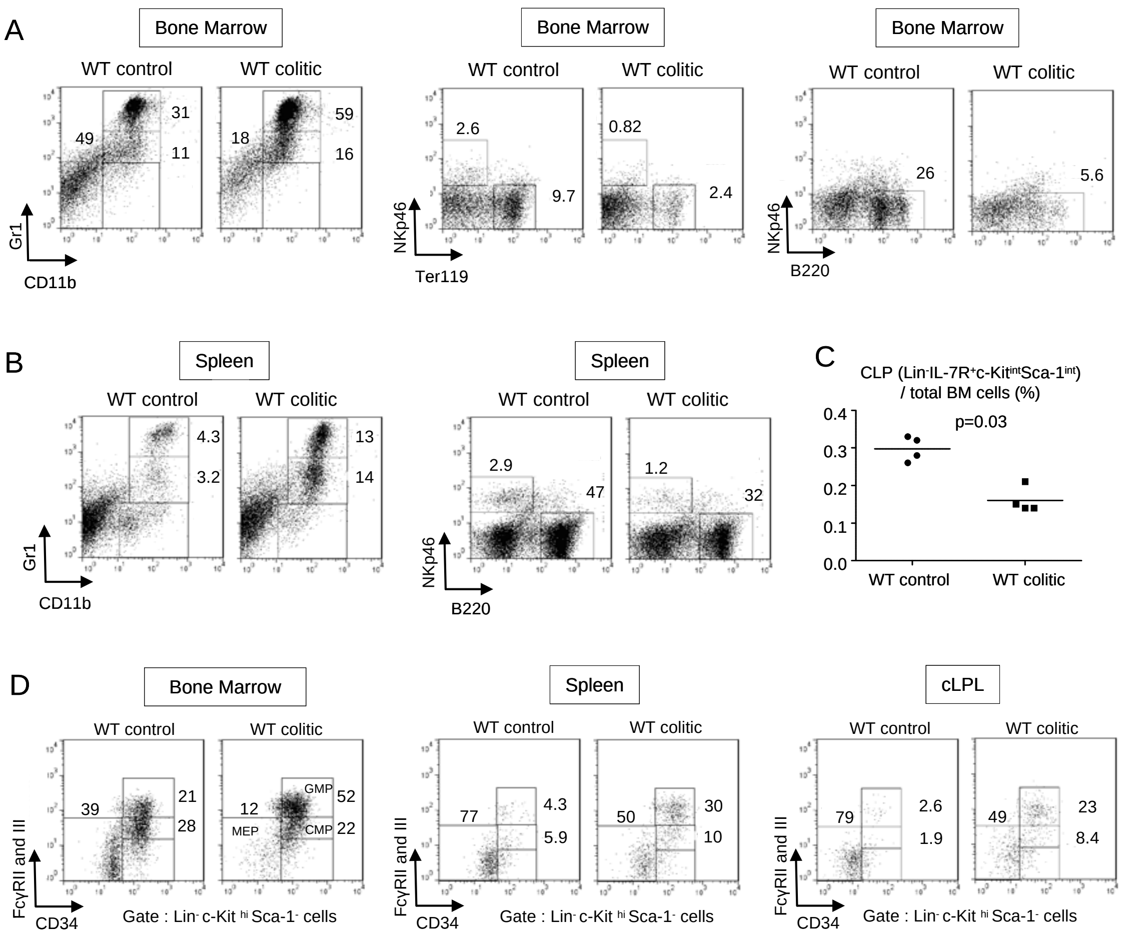

Figure S2. Decreased Lymphopoiesis and Erythropoiesis But Increased Myelopoiesis during Colitis in RAG-Competent Mice, Related to Figure 2

Colitis was induced in WT C57BL/6 mice after infection with *Helicobacter hepaticus* and treatment with anti-IL10R (A-D). Mice were killed at week 4 (WT colitic) and compared with unmanipulated WT mice (WT control).

(A) Representative BM FACS staining of neutrophils ($CD11b^{+}Gr1^{hi}SSC^{hi}$), inflammatory monocytes ($CD11b^{+}Gr1^{int}SSC^{lo}$), NK cells ($NKp46^{+}$), erythroid cells ($Ter119^{+}$) and B cells ($B220^{+}$).

(B) Representative spleen FACS staining of neutrophils, monocytes, NK cells and B cells. Samples were gated on nucleated cells.

(C) Percentages of CLP ($Lin^{-}IL\text{-}7R^{+}Sca\text{-}1^{int}c\text{-}Kit^{int}$ cells) among total BM cells. Each point represents an individual mouse and horizontal bars represent group means.

(D) Representative FACS staining of CMPs, MEPs and GMPs in the BM, spleen and colon. Data are representative of two independent experiments (n= 4 mice per group).

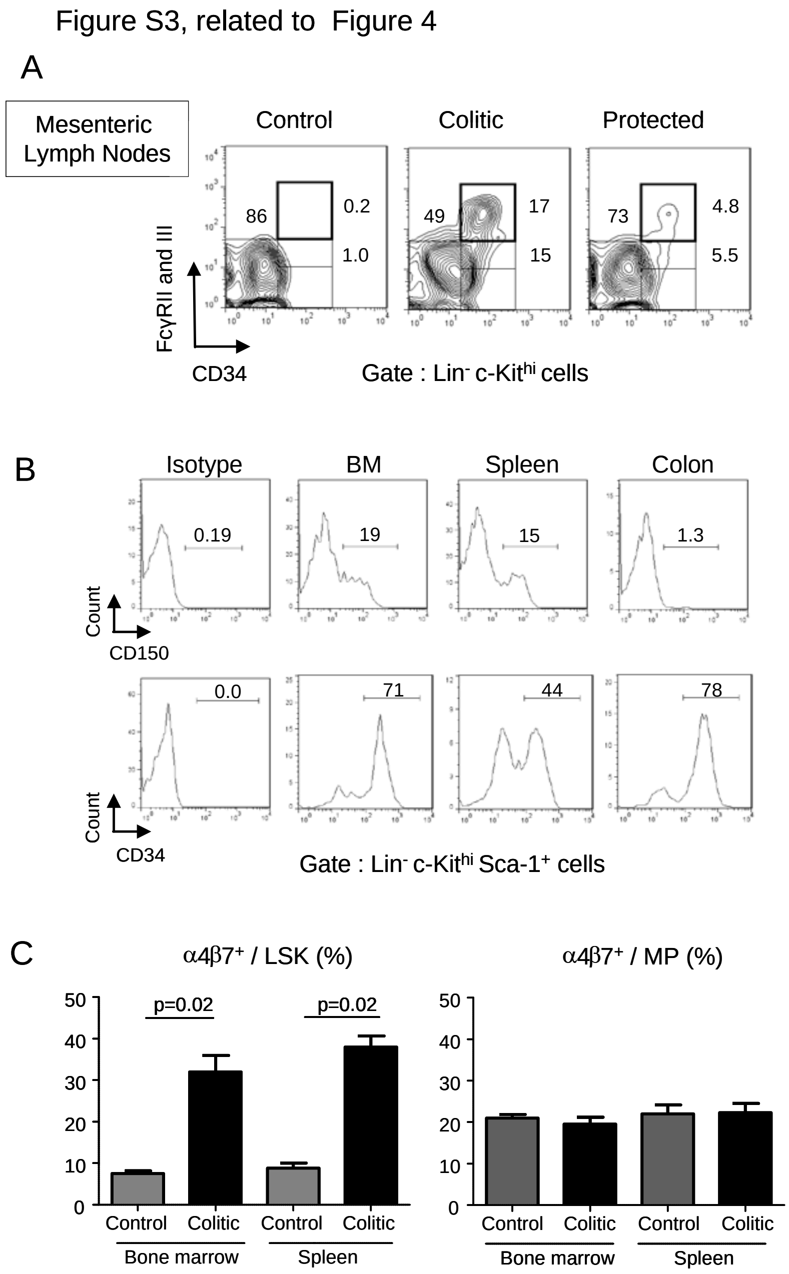

Figure S3. GMP Accumulation in the MLN of Colitic Mice and Differential CD150/CD34 Expression on LSKs in BM, Spleen and Colon, Related to Figure 4

Rag1$^{-/-}$ mice received either CD4^{+}CD45RB^{hi} T cells alone (colitic) or in combination with CD4^{+}CD25^{+} T reg cells (protected), or were untreated (control). Transferred mice were killed 8 weeks after transfer.

(A) Representative staining of CMPs, MEPs and GMPs in the MLN.

(B) CD150 expression (upper panel) and CD34 expression (lower panel) by LSKs in the BM, spleen and colon of colitic mice.

(C) α4β7 integrin expression by LSKs (left panel) and myelo-erythroid progenitors (MP: Lin^{-}c-Kit^{+}Sca-1^{-} cells, right panel).

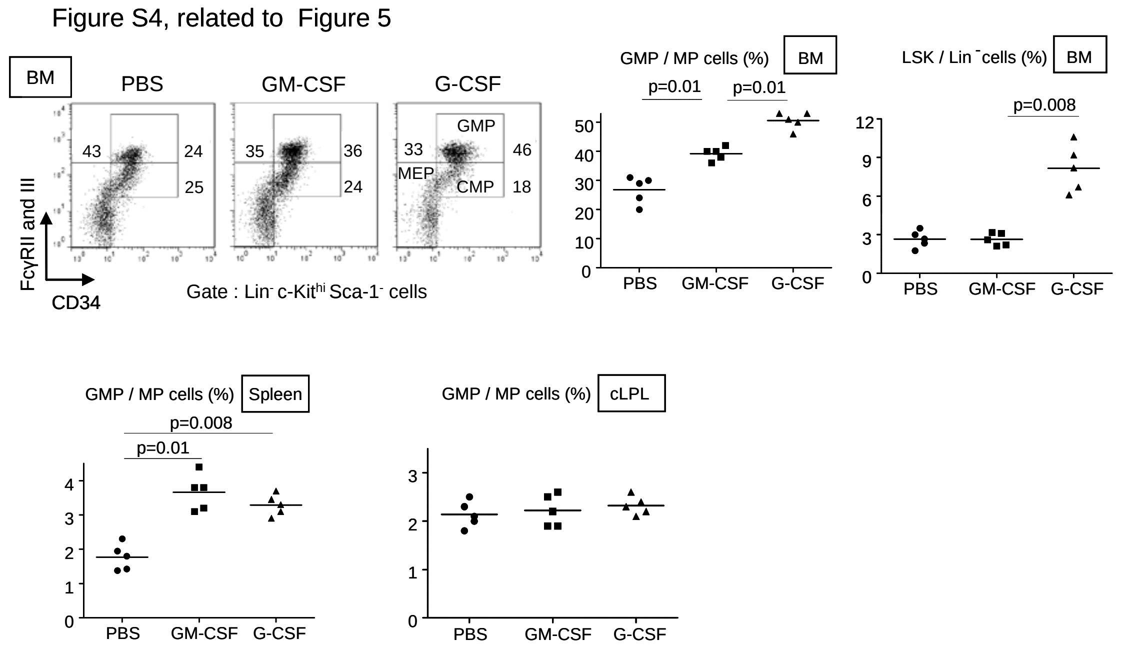

Figure S4. Recombinant GM-CSF and G-CSF Have Similar Effects on GMP Numbers in the BM, Spleen and Colon, Related to Figure 5

C57BL/6.*Rag1*$^{-/-}$ mice were injected daily with G-CSF or GM-CSF or PBS for 3 days and killed 24 hours after the last injection. BM cells, splenocytes and cLPL were harvested and stained for Lin, Sca-1, c-Kit, CD34, FcγRII-III. Myeloid progenitors (MP) are Lin^{-}Sca-1^{-}c-Kit^{hi} cells. Statistical significance was determined using a Mann Whitney test.

Figure S5, related to Figure 6

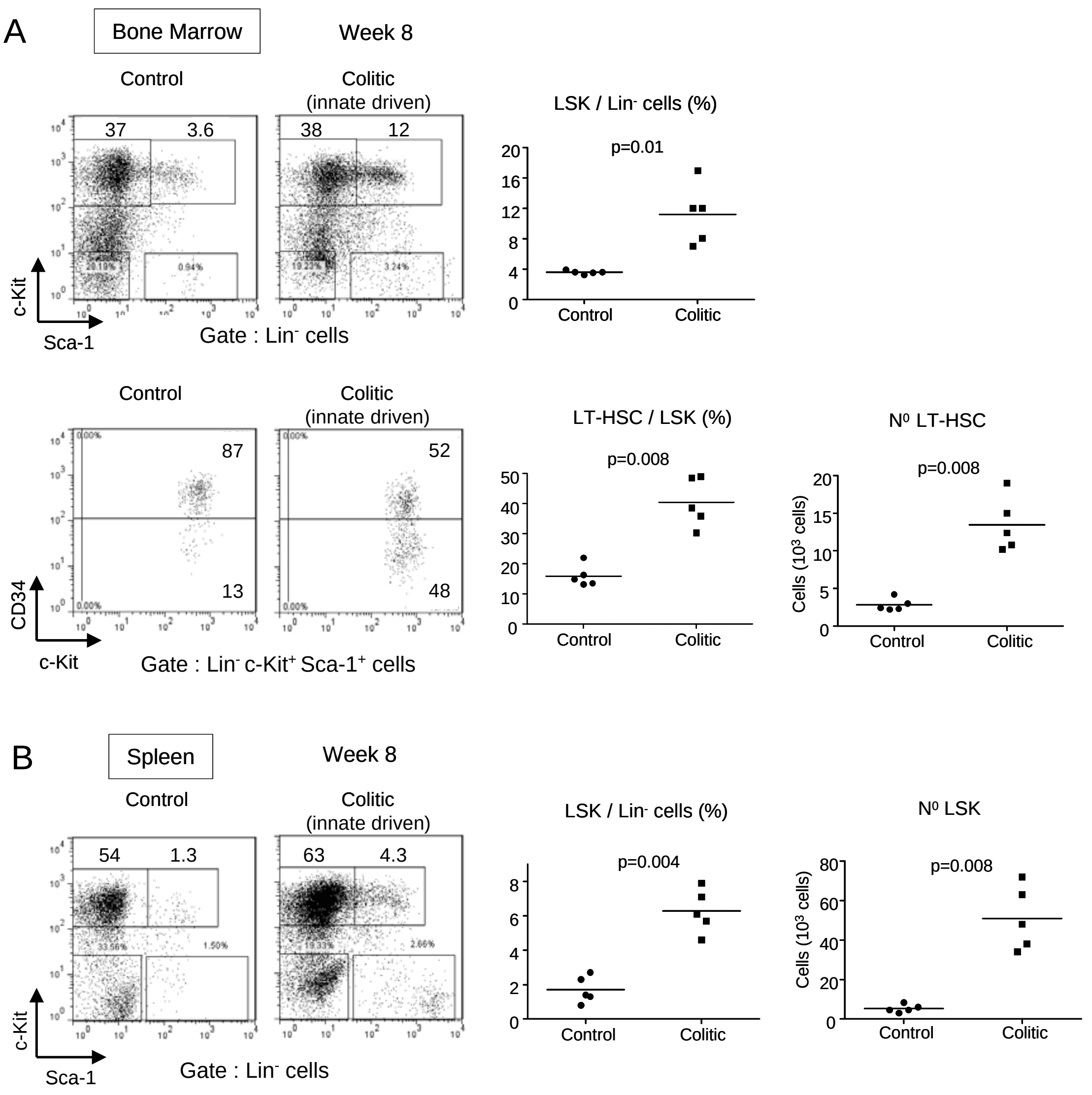

Figure S5. LSK and LT-HSC Are Increased during Innate Cell Driven Colitis, Related to Figure 6

Innate-driven colitis was induced by *Helicobacter hepaticus* infection of 129SvEv.*Rag2*$^{-/-}$ mice. Mice were killed 8 weeks (colitic) after infection and compared to untreated *Rag2*$^{-/-}$ mice (control).

(A) Percentages of LSKs (upper panel) and percentages and absolute numbers of CD34^{-}LT-HSCs (lower panel) in the BM.

(B) Percentages and absolute numbers of LSKs in the spleen. Data are representative of two independent experiments, each point represents an individual mouse and horizontal bars represent group means. Statistical significance was determined using a Mann Whitney test.

Figure S6, related to Figure 7

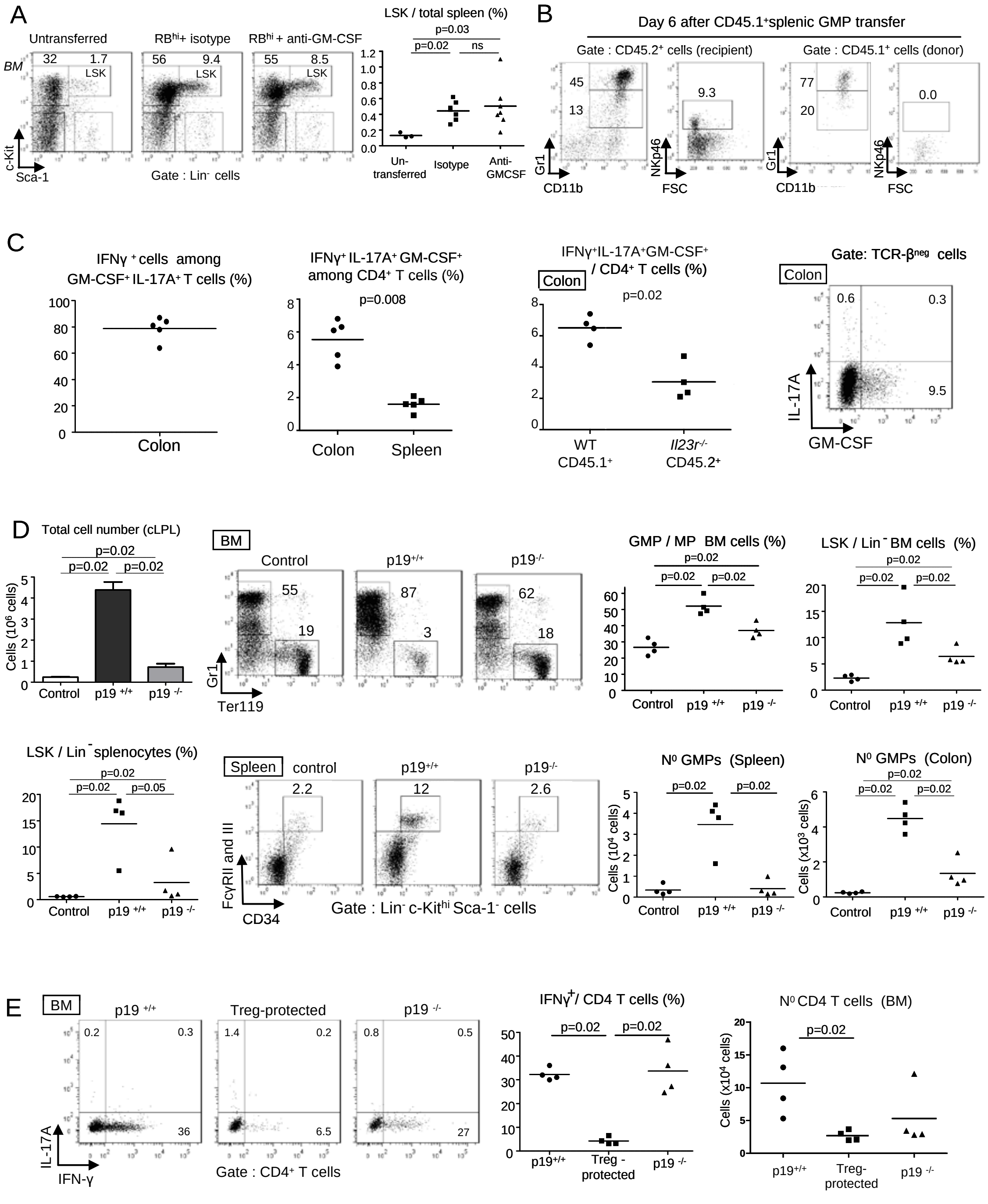

Figure S6. GM-CSF Is Not Required for LSK Increases in the BM and Spleen during T cell Induced Colitis (A), and GMP Transfer Gives Rise to Inflammatory Myeloid Cells during Ongoing Colitis (B), Related to Figure 7

(**A**) *Rag1*$^{-/-}$ mice transferred with CD4^{+}CD45RB^{hi} T cells were injected two times per week with either anti-GM-CSF or isotype control, killed 8 weeks after T cell transfer and compared with untransferred control mice. Representative LSK staining in the BM (left panel) and percentages of LSKs among total splenocytes (right panel). Data are representative of two independent experiments.

(**B**) CD45.2^{+} *Rag1*$^{-/-}$ mice that had received CD4^{+}CD45RB^{hi} T cells 14 days before were injected i.v. with 1x10^{4} CD45.1^{+} splenic GMPs (Lin^{-}Sca1^{-}cKit^{hi}CD34^{+}FcγRII/III^{hi}) sorted from the spleen of colitic mice. Representative staining for myeloid cells (left) and NK cells (right) gated on CD45.2^{+} recipient cells (upper panel) and CD45.1^{+} donor cells (lower panel) in the spleen are depicted. Data are representative of two independent experiments (n= 4 mice in each experiment).

(**C**) *Rag1*$^{-/-}$ mice transferred with WT naïve T cells were killed 6 wk after T cell transfer. cLPL and splenocytes were harvested and restimulated 4 hours with PMA/ionomycin/Brefeldin A. Cells were first stained with live/dead stain and anti-TCR-β and then fixed, permeabilized and stained for intracellular cytokines.

In the mid-right panel, *Rag1*$^{-/-}$ mice were cotransferred with 1:1 mixtures of CD45.1^{+} (WT) and CD45.2^{+} (*Il23r*$^{-/-}$) CD4^{+}CD45RB^{hi} T cells, as described in Figure 7C and in (Ahern et al., 2010), to assess if IL-23 has a cell intrinsic effect on triple producer T cell differentiation (IFN-γ^{+}IL-17A^{+}GM-CSF^{+}). 4 wk after T cell transfer, IL-17A, IFN-γ and GM-CSF levels in colonic CD4^{+} T cells were assessed.

(**D**) *Rag1*$^{-/-}$ *Il23p19*$^{-/-}$ (p19$^{-/-}$) and *Rag1*$^{-/-}$ (p19$^{+/+}$) mice were injected with CD4^{+}CD45RB^{hi} T cells and killed 6 wk after T cell transfer and compared with untransferred *Rag1*$^{-/-}$ control mice. As described in (Hue et al., 2006), T cell transferred *Rag1*$^{-/-}$ *Il23p19*$^{-/-}$ mice had mild colonic inflammation and low leukocyte infiltration (upper left panel) while *Rag1*$^{-/-}$ mice were severely colitic. BM cells were stained for Gr1 and Ter119. BM cells, splenocytes and cLPL were stained for Lin, Sca-1, c-Kit, CD34, FcγRII/III. Of note untransferred *Rag1*$^{-/-}$ *Il23p19*$^{-/-}$ and *Rag1*$^{-/-}$ had similar myeloid progenitor and mature myeloid cell numbers in BM and periphery (data not shown).

(**E**) *Rag1*$^{-/-}$*Il23p19*$^{-/-}$ and *Rag1*$^{-/-}$ mice received CD4^{+}CD45RB^{hi} T cells alone ("p19$^{-/-}$" and "p19$^{+/+}$" respectively) or *Rag1*$^{-/-}$ mice received CD4^{+}CD45RB^{hi} T cells in combination with CD4^{+}CD25^{+} T reg cells ("Treg protected"). 6 wk after T cell transfer BM cells were harvested and stained for IFN-γ production by T cells as described in (**C**).

In **A**, **C**, **D**, **E**, each point represents an individual mouse and horizontal bars represent group means. Statistical significance was determined using a Mann Whitney test.

Supplemental Experimental Procedures

Helicobacter hepaticus Dependent Induction of Colitis

For induction of innate immune colitis by *Helicobacter hepaticus*, 129SvEv.Rag2$^{-/-}$ mice were infected by oral gavage three times on alternate days with $5x10^7$–$2x10^8$ CFU *H. hepaticus*, as described in (Buonocore et al., 2010). Where indicated, mice were injected one time per week with 1mg of anti-IFN-γ (AN-18), starting from the first day of the infection.

Colitis was induced in WT C57BL/6 mice by infecting with *H. hepaticus* as described above and i.p. injection of 1mg 1B1.2 (anti-IL10R) mAb on days 0, 7, 14, and 21 after *H. hepaticus* infection (Kullberg et al., 2006). Mice were killed 1 week after the last mAb treatment.

FACS

The following monoclonal antibodies (eBioscience, BioLegend or BD Biosciences) were used for flow cytometry analysis of mature leukocytes: anti-Gr1 (clone RB6-8C5), CD11b (M1/70), Ter119, B220 (RA3-6B2), NKp46 (29A1.4), CD4 (RM4-5) and TCRβ (H57-597). For HSPC analysis, cells were stained with antibodies against c-Kit (2B8), Sca1 (D7), IL-7Rα (A7R34), CD34 (RAM34), FcγRII/III (CD16/32, clone 93), CD150 (TC15-12F12.2), anti-α4β7 integrin (DATK32) and antibodies against lineage (Lin) markers including CD3, CD4, CD8α, NKp46, B220, CD11b, CD11c, Gr1, Ter119, FcεRIα. For proliferation analysis, cells were fixed in Fix/Perm buffer (eBioscience) after the surface staining, followed by permeabilization in eBioscience buffer and staining with anti-Ki67 mAb or appropriate isotype control. Flow cytometry was performed with an LSRII (BD) and analyzed with FlowJo software (TreeStar).

Quantitation of Gene Expression using Real-Time PCR

After homogenization of frozen colonic samples, total tissue RNA was purified using RNAeasy kits (QIAGEN). Homogenization was performed using a Fastprep 24 Homogenizer (MP Biomedicals). cDNA synthesis was performed using Superscript III reverse transcription and Oligo dT primers (both from Invitrogen). Quantitative PCR reactions were performed using SYBR green PCR SensiMix (Quantace) and GM-CSF, G–CSF, SCF and HPRT primers (QuantiTect; QIAGEN). cDNA samples were assayed in triplicate using a Chromo4 detection system (MJ Research), and gene expression levels for each individual sample were normalized to HPRT. Mean relative gene expression was determined, and the differences were calculated using the 2ΔC(t) method.
